# Are public hospitals reforming efficiently in West Bank?

**DOI:** 10.1186/s13031-018-0180-y

**Published:** 2018-11-05

**Authors:** Wasim I. M. Sultan, José Crispim

**Affiliations:** 1 School of Administrative Sciences, PPU-Palestine & NIPE-Portugal, Hebron, Palestine; 2 School of Economics and Management, University of Minho, Braga, Portugal

**Keywords:** DEA, Public hospitals, Managerial efficiencies, Program efficiencies, West Bank, Jordan

## Abstract

**Background:**

The structure, function, and capacity of the health care system in the Occupied Palestinian Territories (OPT) had been largely shaped by the complex political history of the country. Since the establishment of the Palestinian Authority in 1994, the reform efforts were subsidized much by the international aids to rebuild the country’s institutional capacity. No previous studies have provided a realistic evaluation of Palestinian achievements in the conduct of public healthcare, we examine the relative productive efficiency of public hospitals (their managers’ success in the usage of resources) during 2010–2015 within West Bank and Jordan. Then, we estimate the efficiency of policies within which managers operate (the program efficiency) across the two countries.

**Methods:**

We employ the Data Envelopment Analysis (DEA) models to distinguish between within-country managerial efficiencies and public policy “program” efficiencies across the two countries. The study follows two key steps, the first step evaluates managerial efficiencies and explores trends in performance within each country. Then, we examine the program efficiencies across the two countries.

**Results:**

Public hospitals improved their year-specific overall efficiency from 75 to 80% in the West Bank and from 78 to 86% in Jordan in 2010 and 2015 respectively. Changes in efficiency are driven by scale effects in West Bank and by managerial enhancements in Jordan. Program efficiency in West Bank outperformed Jordan during 2010–2012, there was no significant difference in mean program efficiencies between the two countries during 2013–2015.

**Conclusions:**

This work addresses a gap in the DEA literature by empirically investigating the efficiency of public hospitals as distinct from program efficiency in a developing country, namely, Palestine. Findings stimulate hospital managers to enhance potential improvements, policymakers to allocate resources, and international donors to focus on the right adoption of new technology to get better benefits from their considerable investments in public hospitals.

## Background

The structure, function, and capacity of the health care system in the Occupied Palestinian Territories (OPT) had been largely shaped by the complex political history of the country [1]. The OPT comprises West Bank, East Jerusalem, and Gaza Strip. The never-ending conflict between the Palestinians and the Israelis seemed to come to an end when the Middle East peace process was settled. Particularly, after the Madrid conference in 1991, then the Oslo Accords in 1993 and the establishment of the Palestinian Authority in 1994. However, ground reality suggests otherwise. Regrettably, the peace process reached a deadlock and failed to resolve the conflict. The Palestinians had limited self-determination and constructed a picture that the two-state solution is unapproachable.

Since 1994, the administration of the public health care providers in West Bank and Gaza Strip was transferred from the Israeli Civil Administration to the Palestinian Ministry of Health. However, the other health providers named: The United Nations Relief and Works Agency for Palestine Refugees in the Near East (UNRWA), the Non-Governmental Organizations NGOs, and for-profit private health organization continued as they had been before [2]. Later, the ongoing reform efforts in rebuilding the providers’ capacity have been largely subsidized by international aids. These external funds are efforts by the international community to resolve the conflict in Palestine-Israel through economic encouragements [3], of which, about 8% is dedicated to the healthcare sector [4].

Although noticeable improvements have been made in the physical capacity of public healthcare providers [5], no previous studies have examined the efficient use of the considerable financial and technical investments in public hospitals. Estimating their managers’ efficiency of and the influence of reform efforts on efficiency provide insights to key decision makers in the provision of public health care services in West Bank. Managers who make decisions on operational practices and policymakers who may influence the operating environment through regulations may benefit from the attended measurement. Further, International donors who subsidize the capacity building of the country’s public hospitals may organize their efforts more efficiently.

The hospital productive efficiency entails the use of minimum input to produce a given level of output. In this work, we separate efficiencies of hospital managers themselves (identified as managerial efficiency in this research project) from the efficiencies of policies within which managers operate (identified as program efficiency) by following the method of Charnes et al. (1981) [6]. Health reform policies may support better use of resources or hamper the ability of the hospitals to transform resources into outputs [7]. Therefore, it is reasonable to consider the accumulated efforts of both hospital managers and policy-makers as reform efforts in delivering health care services across public hospitals.

To measure relative productive efficiency, we compare the observed performance of a hospital with an empirical production frontier based on the best results obtained from a homogeneous group of hospitals. About reform policies, a good understanding of the relative Palestinian achievements in the conduct of public healthcare delivery could be suggestive when considering the comparability with Jordan.

We employ the two basic Data Envelopment Analysis (DEA) models, namely the CCR model developed by Charnes et al. (1981) [6] and the BCC model developed by Banker et al. (1984) [8]. DEA is a methodology that has been successfully used to evaluate the performance of different healthcare markets [9].

This paper builds on the method of Charnes et al. (1981) [6]; we follow their two-key steps procedure, in the first step, we analyze the managerial efficiency of public hospitals within each country, West Bank and Jordan. Then, in the second step, we notionally drop the inefficiencies of the first step, and we run a DEA exercise to evaluate the efficiency of a merged set of all the hospitals from the two countries. The observed inefficiencies in the second step are attributable to differences in programs (reform policies) in each country rather than to hospital management.

### Research problem

A recent report of the World Health Organization (WHO) describes the health care system in the OPT as functioning under pressure of rapid population growth, deteriorating economy and lack of adequate financial support [10]. Therefore, addressing the efficiency of public hospitals in the OPT is an important consideration for managers, policymakers, and international donors [11]. The available literature describes the status of the Palestinian healthcare system, although often highlighting the weaknesses and the political constraints, had inadvertently dropped the historical associations with Jordan. Further, doesn’t provide performance measures from a realistic point view to judge the Palestinian achievements in respect of healthcare delivery.

Hamdan & Defever (2002) [2] identified the unique characteristics of the transitional settings such as the geographical separation between West Bank and Gaza Strip; the pressure of international donors; and the deteriorating economic conditions among others. Giacaman et al. (2003) [4] analyzed the obstacles that faced reform efforts. Batniji et al. (2009) [12] described the health and well-being conditions in the OPT and the maternal and child health issues. Giacaman et al. (2009) [1] described the distorted and fragmented healthcare system in the OPT. Others provided statistical evidence on the Palestinian healthcare practices and healthcare professionals [5, 13–16]. Finally, in their qualitative study, Shahawy & Diamond (2016) [17] show the negative impact of geographical barriers and military occupation in the OPT on the training of the Palestinian medical students.

To meet the information needs of public authority and international donors, we provide a realistic benchmark to judge the country’s relative performance concerning public healthcare delivery in West Bank. We pay attention to performance in West Bank and Jordan from two perspectives, the managerial practice perspective within the country and the policy perspective (programs) across the two countries. Therefore, the study goes to answer the following research question: as compared with Jordan, are the public hospitals reforming efficiently in West Bank?

### The comparability between West Bank and Jordan

On 24 April 1950, the “The Emirate of Trans-Jordan” unified with West Bank into one state of “The Hashemite Kingdom of Jordan” that comprised the East and the West Banks of the River Jordan. During 1950–1988 the public health care system in West Bank evolved as part of the public healthcare system in Jordan. Although administered by the Israeli Civil administration during 1967–1994, the institutional affiliation of healthcare providers in West Bank was to the Jordanian Ministry of Health during the latter period.

On 31 July 1988, King Hussain of Jordan broke off all the administrative and legal ties with the Israeli-Occupied West Bank [18]. The disengagement decision represents a turning point in a long story of Jordan’s relations with the Palestinians [19], and initiated new perspectives in the strategy and actors of the peace process in the Middle East [20].

The post-disengagement settings allowed for an effective role of the Palestinian Liberation Organization (PLO) to act internationally as the sole legitimate representative of the Palestinians and challenged PLO to shoulder the Palestinian responsibilities [21]. Even though, the historical associations between Jordan and the Palestinians brand Jordan’s stake in the Palestinians’ life as unavoidable [22]. Roughly, to date, around one-half of the Jordanian population is of Palestinian ancestry [21, 23].

Examples of interrelations are numerous: the Jordanian curriculums, legislation, and the healthcare regulations continued to be applied in West Bank in the early nineties. Further, as well as many other sectors, the Jordanian healthcare institutions were the only recognized boards in the Palestinian healthcare markets. To date, the Palestinian Medical Association in Ramallah is part of the Jordanian Medical Association in Amman. The − 250 beds- hospital “Al Makkassed” in East Jerusalem is still recognized as a teaching hospital by the Jordanian Medical Board [24]. Another public − 275 beds- hospital in Hebron which is officially called “Hebron Governmental Hospital” is still recognized among the public as “Alia” after the name of the daughter of King Hussain “Princess Alia.” The same observation goes to “Al Hussain Hospital” in Bethlehem. Finally, bearing in mind that medical professionals are key decision-makers in healthcare delivery and their institutional background has impacts on their decisions, the proportion of accredited Palestinian specialists by the Jordanian Medical Council is not insignificant.

### The measurement of relative efficiency in the health care context

The question of measuring productive efficiency and its importance for the economic policymakers was best stated sixty years ago in the seminal work of Farrell (1957) [25]. Farrell succeeded to solve the problem of measuring productive efficiency by combining the measurement of multiple inputs into a single satisfactory measure of efficiency. Farrell’s method to estimate productive efficiency compares the observed performance of an organization or industry with an empirical production frontier based on the best results obtained in practice.

Charnes et al. (1978) [26] operationalized Farrell’s approach and introduced the Data Envelopment Analysis (DEA) methodology to measure the efficiency of a homogeneous group of Decision-Making Units (DMUs). DMU is any productive organization “from a small shop to a hospital to a whole economy.” DEA is a nonparametric linear programming-based method for performance evaluation where multiple performance dimensions exist [27]. The method develops best performers frontier and assigns efficiency score for non-frontier hospitals according to their distance to the efficient frontier. In DEA literature, the measurement of efficiency for performance improvement could be tackled from two perspectives; the input contraction (input-oriented DEA models) and the output expansion (out-put oriented DEA models).

The basic DEA model, known as the DEA-CCR model in the literature, assumes Constant Returns to Scale (CRS) in hospital operations. In practice, not all DMUs operate at an optimal scale. Førsund & Hjalmarsson (1974) [28] introduced the decomposition of Farrell’s original measure of productive efficiency into separate scale efficiency and technical efficiency. Then, using the piece-wise linear frontier, the decomposition was considered by Banker et al. (1984) [8] in the DEA-BCC model which accounts for Variable Returns to Scale (VRS).

DEA is the preferred method when evaluating health care providers as it has many advantages: (1) DEA is a non-parametric method and doesn’t require a prescribed functional form. (2) Can handle multiple variables and produce a single measure of efficiency. (3) Each hospital is compared with every other hospital in the sample to estimate the best performers frontier. (4) It avoids the need for prices or prior assumptions of weights [29, 30]. Due to the multiple-product nature of hospitals and the multiple-value of various stakeholders, hospitals received the most research attention in DEA empirical works [31–33]. One of the first applications of DEA was to assess the efficiency of policies within which managers work as separate from the efficiencies of the managers themselves [6].

Based on literature review, Hollingsworth (2008) [30] found that researchers applied DEA to compare the efficiency of hospitals across countries to gain insights into the efficiency of different means of healthcare delivery. It has often been used to evaluate the outcomes of care providers and health reforms [34, 35]. A recent theme in DEA literature evaluates the health care systems across countries [36, 37].

## Methods

### Sample and data

The relevant data in West Bank and Jordan was extracted from the annual health reports published by the Palestinian Ministry of Health (PMoH) and the Jordanian Ministry of Health (JMoH) respectively. The two ministries of interest publish the annual statistical reports on a yearly basis, and both reports are structured alike. The published raw data reflects the real quantities of the operational data among the working hospitals.

We analyzed two nationally representative sets of public hospitals during 2010–2015. Out of 13 public hospitals in West Bank, the analysis included 11 hospitals (66 observations), they utilize 1377 beds which make up 97.4% of the total public hospital beds in West Bank. We excluded a newly established hospital and another psychiatric hospital. Further, the public hospitals in the Gaza Strip are excluded. The relevant data of hospitals working in Gaza Strip is unreliable due to the Palestinian internal conflict since 2006 which escalated with the split of the Palestinian Authority into one government in Gaza and another in West Bank. Therefore, the scope of this papers is to examine efficiency in West Bank [11].

As for Jordan, out of 30 public hospitals, we analyzed 22 hospitals during 2010–2015 (132 observations), three psychiatric hospitals, two obstetric hospitals, and one pediatric hospital were excluded. The excluded hospitals don’t comply with the common input-output measures applied in this study. Then, to avoid bias in our judgment on performance in West Bank, we excluded the largest two hospitals in Jordan as they are three times larger than any hospital in West Bank [24, 38]. Table 1 illustrates the sample characteristics during 2015.

**Table 1:** Public Hospitals in Jordan and West Bank 2015

22 public hospitals in Jordan						11 public hospitals in WB		
	Hospital	Beds		Hospital	Beds		Hospital	Beds
J01	AL-Zarqa	496	J12	AL-Ramtha	110	P01	Yatta	36
J02	Princess Basma	202	J13	Princess Raya	94	P02	Salfit	50
J03	Prince Faisal	178	J14	Ghor AL-Safi	82	P03	Jericho	54
J04	Jarash	116	J15	Mua’th Bin Jabal	75	P04	Nablus/Watani	55
J05	AL-Hussein/Salt	152	J16	Queen Rania	72	P05	Qalqillya	58
J06	Dr. Jameel Totanji	140	J17	AL-Mafraq	70	P06	Tulkarm	117
J07	Ma’an	131	J18	AL-Yarmouk	67	P07	Beit Jala (Hussain)	131
J08	AL-Iman	130	J19	AL-Shuneh (South)	60	P08	Jenin	163
J09	AL-Karak	165	J20	Abu - Obaidah	60	P09	Nablus/Rafidia	200
J10	AL-Nadeem	120	J21	Eiman\ma’di	58	P10	Ramallah	238
J11	Prince AL-Hussein	120	J22	Princess Salma	38	P11	Hebron (Alia)	275

Despite the advantages (mentioned before) of using DEA to evaluate healthcare providers, the pitfalls in DEA methodology should be managed carefully [39]: (1) The state of homogeneity among hospitals, all the investigated hospitals are general hospitals owned and administered by the government, they provide primary and secondary care. However, in Jordan and West Bank, tertiary healthcare is provided by non-public hospitals. (2) The number of inputs and outputs when compared with the number of hospitals. Golany & Roll (1989) suggested a rule of thumb that the number of DMUs is, at least, twice the number of inputs and outputs [40]. To achieve this requirement, we followed Boussofiane et al. (1991) [41] and treated each hospital in each year as an observation and then scored all the observations simultaneously [41]. (3) The clear purpose of measurement applied in this research project, to disentangle the program efficiency from the management efficiency by empirical records obtained from public hospitals. (4) The importance of orientation. The public hospitals have the mission of serving the public demand as given and must manage their limited resources accordingly. Hence, when seeking efficiency assessments, it is recommended to employ the input-oriented DEA models with the aim of input minimizing given a certain level of outputs [42]. We applied the DEA-CCR and the DEA-BCC models to score the overall efficiency, managerial efficiency, and scale efficiency [8, 26].

### The production model of public hospitals

There is no standard set of input-output measures in the DEA literature to analyze the efficiency of hospitals [43]. The fundamental principle, to identify variables, is to have a clear understanding of the “process” being evaluated among hospitals [44]. We used input-output measures that represent the efficiency of access to health care services across hospitals from a productive perspective (technical efficiency) rather than from an economic perspective (economic efficiency). The applied input-output mix in this research project had been used by many researchers in DEA literature [45, 46]. It is worth noting that this mix is a top priority for both managers and policy makers to improve access to health care services in an unstable context, namely, Palestine.

We used output measures that represent the level of social orientation in public health, measures gauge the benefits achieved in respect of three functional areas; admissions (inpatient days), outpatient visits, and emergency services. We used inputs that characterize the employed labor and capital to produce hospital services.

The three main output measures are: (1) inpatient services as measured by the total number of annual care days rather than a number of cases to account for case-mix adjustment [47]; (2) outpatient services as measured by the total number of annual visits [43]; and (3) the emergency services as measured by the total annual number of cases served without admission [45].

On the input side, labor input measures represent three groups of personnel, the full-time employed doctors, the full-time healthcare personnel (e.g., nurses and technicians in para-medical departments), and the full-time administrative personnel [43, 48]. The number of hospital beds represents the capital input measure [49]. Table 2 presents the distribution of input-output variables used during the study period from 2010 through 2015.

**Table 2:** Distribution of input-output measures during the period 2010–2015

Period		Input measures				Output measures		
		Hospital beds (X1)	Doctors FTEs^a^ (X2)	Health FTEs (X3)	Admin. FTEs (X4)	Inpatient days (Y1)	Outpatient visits (Y2)	EM. visits (Y3)
West Bank *N* = 66								
2010–15	Mean	116	48	180	75	36,765	40,540	63,632
	St. Dev.	9	3	12	3	3380	3262	3871
Jordan *N* = 132								
2010–15	Mean	113	76	270	74	25,158	105,283	75,598
	St. Dev.	6	6	11	3	1545	6481	4163

^a^FTEs, Full-Time Employees. EM, Emergency

### Within-country analysis: the assessment of overall and managerial efficiencies

This step of analysis applies the basic DEA models; the CCR model [26] and BCC model [8], the CCR model estimates the overall efficiency and the BCC model estimates the managerial efficiency. Then, in the next step, we apply a procedure developed by Charnes et al. (1981) [6] to estimate program efficiency. The type of information drawn from these models achieves our research objective.

When longitudinal data is available for hospitals, different ways of analyses can provide alternative views on hospital performance. Window Analysis and DEA-Malmquist Index are known in the DEA literature to approach the efficiency changes over time [50, 51]. These methods elaborate further on the performance changes of the individual hospital. However, we are looking for a realistic indicator to evaluate the relative success of the evolving public hospital services in West Bank in respect of Jordan, rather than to produce individual tables for each hospital.

We conducted a 6-year evaluation of two sets of hospitals from 2010 to 2015. The first set comprises 66 observations in West Bank. The second set comprises 132 observations in Jordan. We applied a procedure that captures the year-specific variability in performance of hospitals [41, 52]. For each country, we treated every hospital as a different DMU every year; the yielded DEA estimates are the observed relative efficiencies compared with best performers over the six years of study within the country. The applied procedure avoids the misleading results of efficiency change between consecutive years when DEA assessment is carried out separately for each year. Since performance measurement is relative, the increase in efficiency of one hospital between consecutive years could be attributed to an actual improvement or attributed to a regress in the efficiency (frontier shift) of the whole set of hospitals. Moreover, the simultaneous assessment of 66 Palestinian observations and 132 Jordanian observations improves the discrimination power of the DEA estimator [44]. The mathematical linear programming formulation of the CCR and BCC models is presented in Appendix A.

It is informative to know whether any inefficiency is the consequence of hospitals’ scale of operations or managerial practices. Scale inefficiency informs how far a hospital is operating from the most productive size for a given input-output mix. To help managers capture the components of inefficient operations, the sequential analysis of the DEA-CCR and the DEA-BCC models allow for the computation of three types of efficiencies [53]:

1. Overall efficiency (OE) as measured by the distance to the DEA-CCR frontier.
2. Pure technical efficiency (identified as managerial efficiency (ME) in this work) as measured by the distance to the DEA-BCC frontier.
3. Scale efficiency (SE) which reflects the proportion of inefficiency caused by the given scale of operations. It is measured by the ratio DEA-CCR score/DEA-BCC score.

### Across country analysis: the assessment of program efficiency

We follow the work of Charnes et al. (1981) [6] to evaluate the program efficiency of public healthcare delivery in West Bank compared to Jordan. An attempt would be valid if the managerial inefficiency within the country is notionally eliminated and all the hospitals are treated as being managerially efficient. This adjustment could be made by moving inefficient hospitals to their frontier within each set of hospitals. As we applied input-oriented DEA-BCC, reducing all the inputs by the estimated inefficiency score will position all the hospitals on the efficient frontier of their country. After these adjustments have been made up, the new DEA-BCC assessment is carried out comprising a merged sample of all hospitals from West Bank and Jordan (66 + 132 = 198 observations). Hence, any new inefficiencies observed will be attributable to the country’s public policy and reform efforts rather than to local hospital management.

## Results and discussion

As we are employing input-oriented DEA models, in this sense, the resulting efficiency score of a given hospital has the following operational information: it is the maximum proportion of input levels the hospital, if efficient, should spend to secure at least its current output level. For example, efficiency score of 0.85 for a hospital means that this hospital should have used only 85% of what it had expended or could save 15% of its resources while producing the same observed output levels. Inefficient hospital benchmarks a composite hospital of its reference set to improve performance. Because we are addressing market level rather than hospital level, the technique used to design a reference set at hospital level is out of the scope of this work [41, 54, 55].

The following subsections are organized according to our research objective and convey three main results: (1) Overall and managerial efficiency scores of 66 Palestinian observations; (2) Overall and managerial efficiency scores of 66 Jordanian observations; and (3) the reforming efficiency scores across the two countries.

### Overall efficiency and managerial efficiency of public hospitals in West Bank

**Fig. 1:**
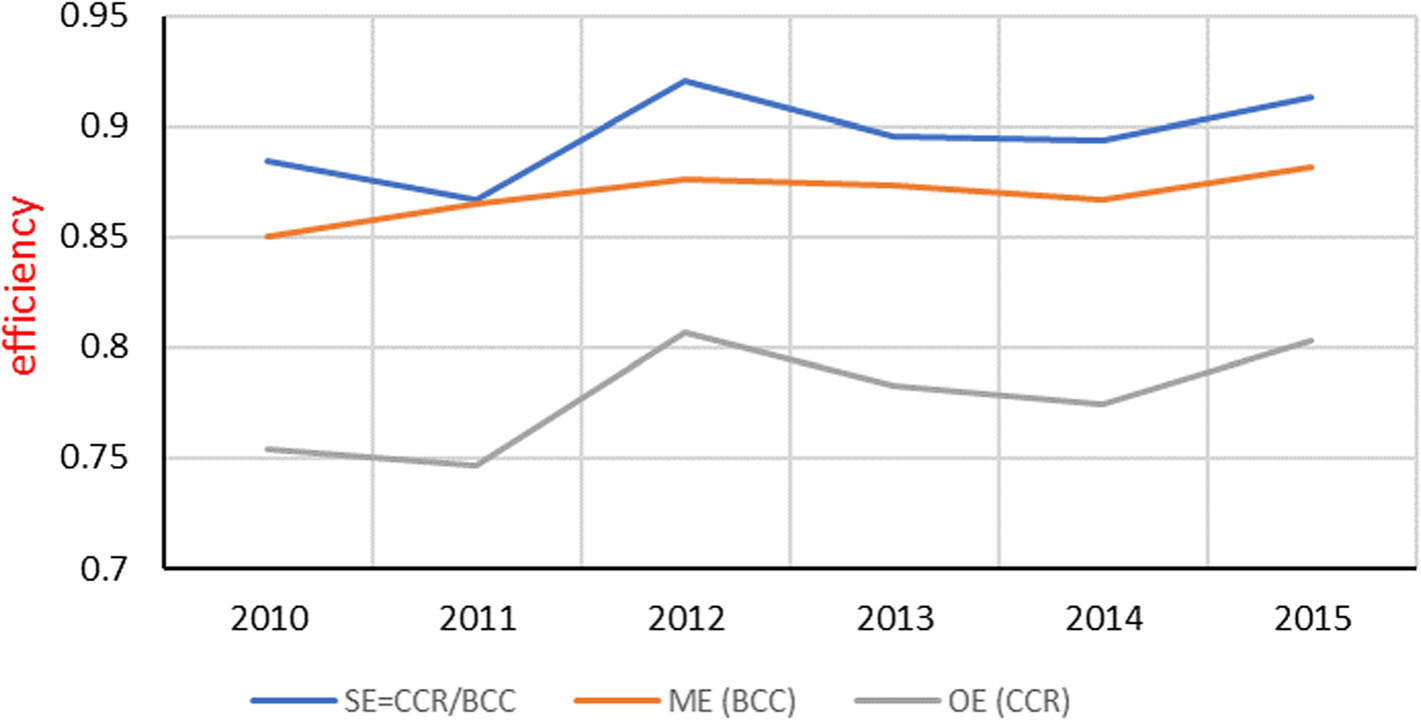
Year-specific means overall efficiencies (OE), managerial efficiencies (ME), and scale efficiencies (SE) of public hospitals – West Bank (2010–2015)

Figure 1 illustrates the Palestinian results presented in Table 3, the year-specific mean efficiency scores of 11 public hospitals. Overall Efficiency scores (OE) represent the relative performance as measured by the radial distance to the best performers among the 66 Palestinian observations. The DEA-CCR scores revealed ten efficient observations (15%) in the overall sense. In this regard, these hospitals are managerially efficient and operating under optimal scale. Managerial Efficiency (ME) shows 20 observations efficient (30%) as given by the DEA-BCC scores, the difference observed in some efficient hospitals is the scale inefficient observations, these are ten hospitals.

**Table 3:** Year-specific means, efficiency scores of hospitals in West Bank and Jordan

West Bank (*N* = 66)	2010	2011	2012	2013	2014	2015^b^	Average Total
Overall efficiency^a^							
Mean efficiency	0.75	0.75	0.81	0.78	0.77	**0.80**	0.78
Efficient hospitals	2	1	2	2	2	**1**	10
Managerial efficiency							
Mean efficiency	0.85	0.87	0.88	0.87	0.87	**0.88**	0.87
Efficient hospitals	4	3	3	3	4	**3**	20
Scale efficiency							
Mean efficiency	0.88	0.87	**0.92**	0.90	0.89	**0.91**	0.90
Efficient hospitals	2	1	2	2	2	**1**	10
Jordan (*N* = 132)	2010	2011	2012	2013	2014	**2015**	Average Total
Overall efficiency							
Mean efficiency	0.78	0.77	0.82	0.76	0.83	**0.86**	0.80
Efficient hospitals	4	0	3	2	7	**8**	24
Managerial efficiency							
Mean efficiency	0.89	0.88	0.90	0.87	0.90	**0.94**	0.90
Efficient hospitals	9	3	6	4	10	**12**	44
Scale efficiency							
Mean efficiency	0.87	0.88	0.91	0.88	0.91	**0.92**	0.90
Efficient hospitals	4	0	3	2	3	**8**	24

^a^Overall efficiency (CCR scores), Managerial efficiency (BCC scores), and scale efficiency (CCR score/BCC score)

^b^The boldface data in 2015, because the year 2015 shapes the possible future improvements

The overall efficiency as given by the year specific mean CCR scores improved during the study period from 75% in 2010 to 80% in 2015. Even though, hospitals in 2015 could produce the observed level of outputs using 20% fewer inputs than they did. It is worth noting the peak “overall efficiency” is 81% in 2012. The large investment of the Palestinian Ministry of Health in installing the Medical Information System in public hospitals during 2011; this may explain progress achieved in efficiency in 2012. The year-specific managerial efficiency (ME) as given by the mean BCC scores shows a slightly positive change from 85% in 2010 to 88% in 2015.

It is useful to witness the parallel shape of scale efficiency (SE) with the overall efficiency (OE). This finding suggests that, during the study period, changes in OE are driven by changes in SE rather than by changes in ME. The mean managerial efficiency (as given by the mean BCC scores) is 88% in 2015 and suggests 12% room for performance improvement, the 12% inefficiency is mainly attributed to poor managerial practices. On the other hand, the 9% scale inefficiency is attributed to poor allocation of resources or technologies [48]. The year-specific individual scores are presented in Table 4 within Appendix B.

### Overall efficiency and managerial efficiency of public hospitals in Jordan

**Fig. 2:**
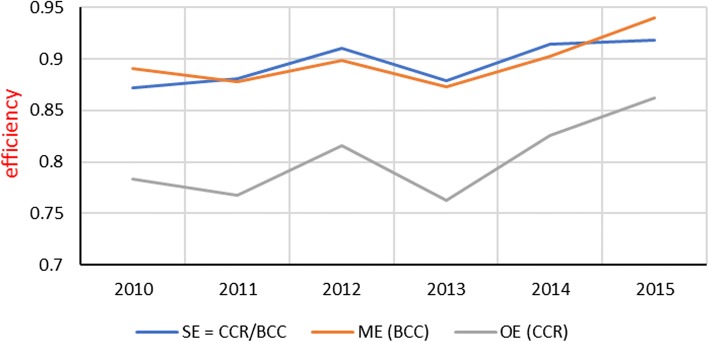
Year specific mean overall efficiencies (OE), managerial efficiencies (ME), and scale efficiencies (SE) of public hospitals - Jordan (2010–2015)

Figure 2 illustrates the results of examining the year-specific mean efficiency scores of 22 Jordanian public hospitals Jordanian hospitals (see Table 3). Efficiency scores represent the relative performance as measured by the radial distance to the best performers among the 132 Jordanian observations.

In the overall sense, the DEA-CCR scores revealed 24 efficient observations (18%). In the managerial sense, the DEA-BCC efficiency scores show 44 observations efficient (33%). The observed difference is the 20 scale inefficient observations.

During the study period, the overall efficiency improved from 78% in 2010 to 86% in 2015. Even though, hospitals in 2015 could produce the observed level of outputs using 14% fewer inputs than they did. Managerial efficiency show**s** 6% possible improvement in 2015 as given by the mean BCC scores; this is mainly attributed to poor managerial practices [48]. On the other hand, the 8% scale inefficiency in 2015 is attributed to poor allocation of resources and technologies among the Jordanian hospitals.

The remarkable result is the consistent progress and a promising trend in managerial efficiency since 2013, from 87% in 2013 to 90% in 2014 then to 94% in 2015 as given by the DEA-BCC scores. Further, the mean year specific of managerial efficiency has a parallel shape with the overall efficiency, which reveals that progress in overall efficiency is driven by gains in managerial efficiency rather than by changes in scale efficiency. Table 5 within Appendix B exhibits the year-specific individual scores of Jordanian public hospitals.

So far, changes in mean overall efficiency scores of public hospitals were shaped differently in West Bank and Jordan during the study period. In West Bank, efficiency progress was driven by improvements in scale efficiency levels. In Jordan, efficiency progress was driven by improvements managerial performance.

### Across country analysis: program efficiency

For comparing the health care reform efforts in West Bank and Jordan, we notionally eliminate the estimated managerial inefficiency of the first step of this study. The basic idea is to drop the inefficiencies attributed to poor managerial practices before carrying out the new assessment. Since all the hospitals have become on the efficient frontier within the country, the new assessment captures new inefficiencies that are attributable to the country’s programs and not management [6].

**Fig. 3:**
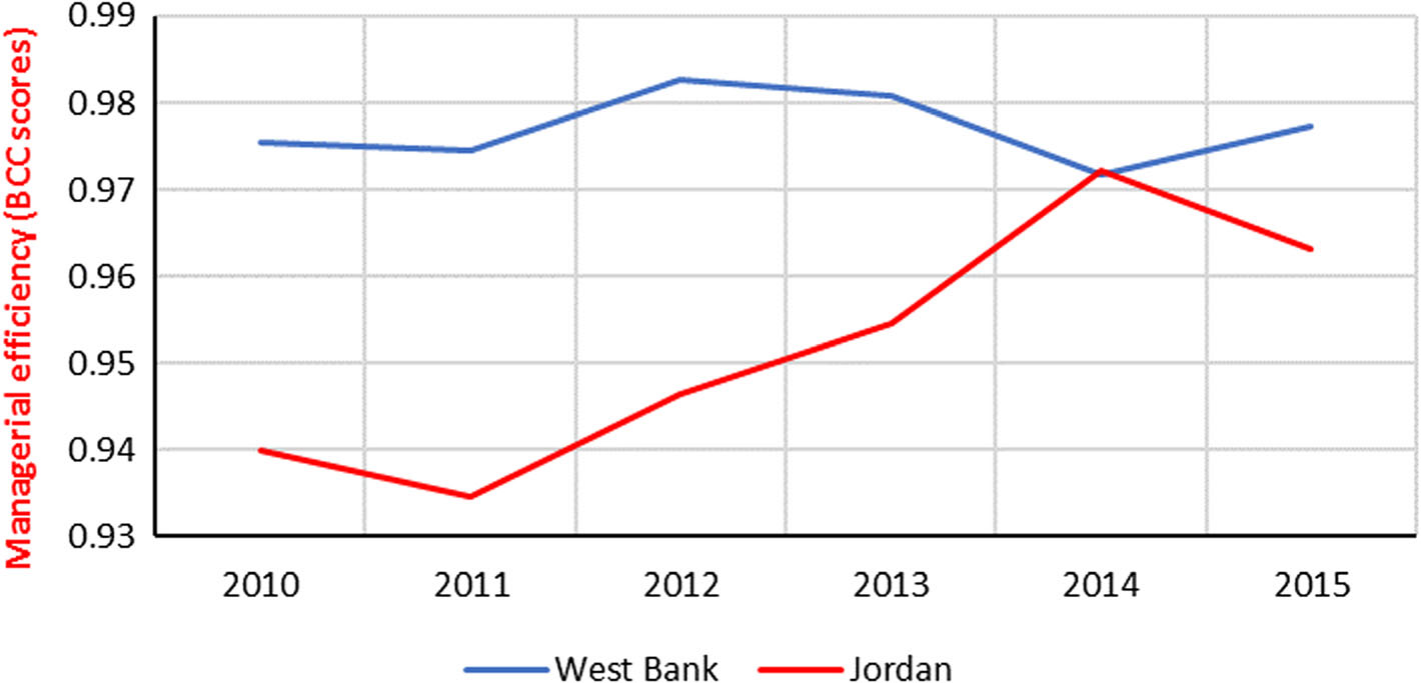
Program Efficiency. Year-specific means, DEA-BCC scores after eliminating the within the country inefficiencies

Figure 3 illustrates program efficiency scores in West Bank and Jordan as given by the DEA-BCC model, Table 6 and Table 7 within Appendix B show the relevant results. Public hospitals’ performance shows differences (0–4%) in mean program efficiency between the hospitals in the West Bank and hospitals Jordan in favor of West Bank.

A single sample T-test was conducted to determine if a statistically significant difference exists between mean program efficiency of Jordanian hospitals and mean program efficiency of Palestinian hospitals. During the first three-years (2010–2012), Jordanian program efficiency reported different mean scores compared to Palestinian program efficiency (*p* < 0.025). For example, in 2010, the difference reported - 4% (M = 0.94, SD = 0.05) in favor of Palestinian programs compared to Jordanian programs t (21) = − 2.78, *p* = 0.001. However, during the second three-years of the study period (2013–2015), the Jordanian program efficiency reported similar mean scores (*p* > 0.025). For example, in 2015, the difference reported – 1.6% (M = 0.96, SD = 0.05) in favor of Palestinian programs compared to Jordanian programs t (21) = − 1.603, *p* = 0.124.

To sum up, this study addresses the relative position of Palestinian healthcare programs in West Bank after thirty years of disengagement with Jordan. In this regard, the accumulated outcomes of the two countries’ programs for public hospital services are compared. Unexpectedly, findings show that the implications of Palestinian programs on efficient hospital performance have the edge over the Jordanian programs during 2010–2012. Saying it another way, in West Bank, the inefficient performance is less attributed to public programs than in Jordan during in the first three years of the study period. However, the Jordanian programs show consistent progress during the years 2011–2014. Therefore, the two countries’ programs have no significant difference in program efficiency means during 2013–2015.

Although all the investigated hospitals are public and don’t compete, it is felt that the market characteristics of each country may impact efficiency. Palestinian Authority applies health insurance schemes that cover most of the population. Therefore, Palestinian public hospitals are asked to meet the created high demand [5]. The average bed occupancy rate was 88% during 2015 in the West Bank while it was 65% in Jordan during the same period that explains higher levels of capacity utilization in West Bank. Previous evidence supports the impact of bed occupancy rate on efficiency [49, 56].

Another reason the estimated efficiency values in West Bank and Jordan may be the external international fund and the considerable amount of financial and technological investments supported by the international donor in Palestinian hospitals [1]. This, in turn, makes a hospital bed more equipped with medical and paramedical devices and becomes more productive. However, in our analysis, we did not include this nontraditional input, further research could be useful to investigate the level of adoption of new technologies in West Bank.

Finally, the working market-structure may influence the efficiency of a hospital. In West Bank, the percentage of public hospital beds of all the beds is 61%. In Jordan, this is 38.7% of all the beds. This could influence people seeking hospital treatment, thus determine the demand and the observed output levels. Further research on the topic to determine the factors that drive the outperformance of Palestinian hospitals to outperform the Jordanian hospitals is useful.

## Conclusions

Using data from 2010 through 2015, we employed the two basic DEA models and analyzed the productive efficiency of public hospital markets within West Bank and Jordan. We analyzed public hospitalization services within the distinct country conditions of the Occupied Palestinian Territories, particularly after thirty years of Jordan’s decision to end the long institutional, legal, and administrative ties that prolonged from 1950 to 1988 with West Bank. After a transition period, the affiliation of the public hospitals in West Bank was transferred to the Palestinian Ministry of Health in 1994. This work makes a major contribution by extending the descriptive literature of historical associations between the Occupied Palestinian Territories and Jordan with a comparative measure of the implications of public reforms on hospital performance.

This study took the Palestinian health literature further by highlighting Jordan’s ongoing stake in the Palestinian healthcare sector. Results surface the significance of operational trade-offs to enhance the productive efficiency of public hospitals by employing realistic operational indicators. Findings provide evidence of the prescribed uneven emphasis of the Palestinian public authorities on the physical capacity [5] and the considerable amount of financial and technological investments [4].

In West Bank, managers of the identified hospitals with poor management as given by the DEA-BCC model should investigate further their managerial practices applied in their hospitals. The Palestinian policymakers can draw from the identified scale inefficiencies an appropriate resource reallocation plan. Since the overall mean efficiency score is inadequate (78%) and scale efficiency drives the year-specific progress during the study period, the operating conditions explore the question of appropriate adoption with technological investments and the adequate managerial efforts to capture the acquired benefits of technology [48].

In Jordan, the average overall efficiency score recorded 80% with a promising trend in 2015. However, the changes over time were driven by managerial improvements and not scale efficiency progress. Despite the similarity in changes in hospital performance, changes were driven differently in West Bank and Jordan. Year-specific improvements were attributed to management in Jordan and attributed to new technologies in West Bank.

Health reforms contributed differently to the achieved levels of efficiency across the two countries. The inefficient performance was less attributed to policies in West Bank than the corresponding effect in Jordan. The Palestinian policy implications on hospital performance have the significant edge over Jordan policies during 2010–2012. In Jordan, the continuous improvements in policies during 2011–2014 positioned the two countries at the same level in 2014 but turned again less supportive in 2015.

Because Egypt administered Gaza Strip during the same period West Bank unified with Jordan, and because of the lack of reliable data of hospitals in Gaza, we don’t investigate the performance of public hospitals in the Gaza Strip. Further, our findings don’t adjust for quality differences within or across the two countries. The scope of our analysis considers only quantities of services provided and resource distribution. Unfortunately, data associated with qualitative information, as in many other developing countries, is not available.

## Appendix A

### The mathematical formulations of Data Envelopment Analysis model

For *n* Decision Making Units DMU with *m* inputs and *s* outputs, The DEA-CCR input oriented envelopment model that builds Constant Returns to Scale CRS efficient frontier has a mathematical linear programming formulation [26]:

1 $$ {\uptheta}^{\ast }=\operatorname{Min}\kern0.5em {\uptheta}_{\mathrm{o}} $$

S.T

$$ {\sum}_{j=01}^{j=n}{\lambda}_j\ {x}_{ij}\le \theta {x}_{io}\kern3.25em i=1,2,\dots, n $$

$$ {\sum}_{j=01}^{j=n}{\lambda}_j\ {y}_{rj}\ge {y}_{ro}\kern4em r=1,2,\dots, s $$

$$ {\lambda}_j\ge 0\kern4em j=1,2,\kern1em ..\kern0.5em ,n $$

Where:

DMUo represents one of the n DMUs under evaluation, and x_io_ and y_ro_ are the i^th^ input and r^th^ output for DMUo, respectively.

The DEA-CCR input oriented envelopment model that builds Variable Returns to Scale VRS efficient frontier requires an additional set of convexity constraint of the dual linear programming algorithm (Eq. 1); the sum of lambdas to be one. The additional constraint is written as Eq. 2 [8]:

2 $$ {\sum}_{j=01}^{j=n}\ {\lambda}_j=1.0\kern16.5em $$

## Appendix B

### Year-specific results of relative efficiency presented in Tables

**Table 4:** Year-specific relative efficiency scores of hospitals in West Bank

Hosp.	DEA-CCR model scores						DEA-BCC model scores					
	2010	2011	2012	2013	2014	2015	2010	2011	2012	2013	2014	2015
P01	1	0.92	1	0.89	0.98	0.97	1	0.99	1	0.99	1	1
P02	0.57	0.66	0.67	0.57	0.58	0.64	0.83	1	0.88	0.88	0.9	0.9
P03	0.49	0.51	0.67	0.63	0.6	0.65	0.84	0.8	0.88	0.85	0.83	0.93
P04	0.97	0.93	0.99	1	1	0.98	1	0.95	1	1	1	0.99
P05	0.88	0.68	0.71	0.78	0.77	0.82	1	1	0.85	0.89	0.87	0.87
P06	0.62	0.63	0.69	0.72	0.69	0.69	0.67	0.71	0.74	0.76	0.73	0.73
P07	0.73	0.72	0.82	0.73	0.65	0.68	0.85	0.79	0.84	0.75	0.67	0.69
P08	0.75	0.78	0.77	0.65	0.71	0.78	0.77	0.8	0.77	0.7	0.76	0.78
P09	0.64	0.64	0.76	0.76	0.75	0.78	0.66	0.66	0.76	0.79	0.78	0.81
P10	0.64	0.74	0.8	0.88	0.79	0.84	0.73	0.82	0.92	1	1	1
P11	1	1	1	1	1	1	1	1	1	1	1	1
Ave.	0.75	0.75	0.81	0.78	0.77	0.80	0.85	0.87	0.88	0.87	0.87	0.88

Overall (CCR scores), pure technical (BCC scores) and scale (CCR score/BCC score) efficiencies

**Table 5:** Year-specific relative efficiency scores of hospitals in Jordan

Hosp.	DEA-CCR model scores						DEA-BCC model scores					
	2010	2011	2012	2013	2014	2015	2010	2011	2012	2013	2014	2015
J01	1	0.79	0.97	1	0.9	1	1	0.86	0.97	1	0.9	1
J02	1	0.95	1	1	1	1	1	0.97	1	1	1	1
J03	1	0.97	1	0.98	1	1	1	0.97	1	0.98	1	1
J04	0.83	0.78	0.66	0.59	0.72	0.77	0.83	0.79	0.66	0.61	0.73	0.82
J05	0.81	0.59	0.78	0.65	0.71	0.74	0.81	0.59	0.8	0.69	0.74	0.78
J06	0.84	0.88	0.91	0.84	0.9	1	0.85	0.9	0.92	0.85	0.9	1
J07	1	0.95	0.9	0.81	1	0.94	1	0.98	0.92	0.85	1	0.96
J08	0.83	0.68	0.74	0.67	0.79	0.9	0.83	0.68	0.75	0.72	0.8	0.94
J09	0.62	0.66	0.79	0.72	0.58	0.51	0.66	0.7	0.85	0.8	0.63	0.56
J10	0.73	0.72	0.92	0.8	1	1	0.78	0.76	0.94	0.84	1	1
J11	0.12	0.69	0.69	0.62	0.78	0.75	0.59	0.84	0.73	0.68	0.81	0.81
J12	0.65	0.73	0.85	0.73	1	0.85	0.73	0.8	0.86	0.8	1	0.89
J13	0.74	0.78	0.7	0.66	0.8	0.87	0.83	0.87	0.81	0.78	0.89	0.98
J14	0.89	0.91	0.93	0.91	0.95	0.9	0.97	0.98	1	0.98	1	1
J15	0.87	0.68	0.76	0.76	0.78	1	1	0.88	0.87	0.89	0.87	1
J16	0.51	0.6	0.51	0.56	0.48	0.46	0.92	1	0.88	1	0.9	0.98
J17	0.84	0.87	0.91	0.96	1	1	0.9	0.96	0.96	0.98	1	1
J18	0.84	0.87	0.89	0.8	1	0.91	0.89	0.91	0.92	0.91	1	0.95
J19	0.78	0.49	0.54	0.52	0.5	0.77	1	0.88	0.92	0.85	0.86	1
J20	0.96	0.95	1	0.98	0.8	1	1	0.99	1	0.99	0.83	1
J21	0.78	0.77	0.88	0.72	0.87	0.88	1	1	1	1	1	1
J22	0.6	0.58	0.62	0.5	0.6	0.72	1	1	1	1	1	1
Ave.	0.78	0.77	0.82	0.76	0.83	0.86	0.89	0.88	0.90	0.87	0.90	0.94

**Table 6:** Program efficiency scores - West Bank

Post adjustment DEA-BCC assessments.						
Hosp.	2010	2011	2012	2013	2014	2015
P01	1	1	1	1	1	1
P02	0.99	1	1	0.97	0.95	0.97
P03	0.94	0.97	0.98	0.98	0.96	0.96
P04	0.92	0.94	0.97	1	1	0.98
P05	1	1	1	1	1	1
P06	0.99	1	1	1	1	0.99
P07	0.98	0.98	1	1	1	1
P08	0.99	0.95	0.99	0.99	0.99	1
P09	1	1	0.96	0.93	0.95	0.94
P10	0.93	0.95	0.93	0.93	0.84	0.91
P11	0.99	0.93	0.98	0.99	1	1
Average	0.98	0.97	0.98	0.98	0.97	0.98

**Table 7:** Program efficiency scores - Jordan

Post adjustment DEA-BCC assessments.													
Hosp.	2010	2011	2012	2013	2014	2015	Hosp.	2010	2011	2012	2013	2014	2015
J01	1	1	1	1	1	1	J12	0.93	0.9	0.98	0.87	1	0.95
J02	1	1	1	1	0.94	1	J13	0.91	0.95	0.92	0.93	0.94	0.96
J03	1	0.99	1	1	1	1	J14	0.92	0.91	0.93	0.98	1	0.91
J04	0.96	0.98	1	0.95	1	0.85	J15	0.93	0.94	0.96	1	1	1
J05	1	1	0.95	0.96	0.97	0.92	J16	0.98	1	0.94	1	0.97	1
J06	0.87	0.87	0.84	0.84	0.9	1	J17	0.91	0.85	0.93	0.95	0.9	1
J07	0.9	0.81	0.84	0.93	0.92	0.85	J18	0.91	0.97	0.97	0.97	0.92	0.94
J08	0.98	0.98	1	0.98	1	0.99	J19	1	0.91	0.88	0.95	1	1
J09	0.86	0.81	0.9	0.94	0.91	0.9	J20	0.96	0.98	1	1	0.91	1
J10	0.87	0.92	1	0.86	1	1	J21	1	0.9	0.98	1	1	0.97
J11	0.86	0.96	0.92	0.89	0.99	0.95	J22	0.93	0.93	0.88	1	0.99	1
							Average	0.94	0.93	0.95	0.95	0.97	0.96

## Abbreviations

BCC: A Model developed by Banker Charnes and Cooper 1984
CCR: A model developed by Charnes, Cooper, and Rhodes 1978
CRS: Constant Returns to Scale
DEA: Data Envelopment Analysis
DMU: Decision Making Unit
JMoH: Jordanian Ministry of Health
ME: Managerial Efficiency
NGOs: Non-Governmental Organization
OE: Overall Efficiency
OPT: Occupied Palestinian Territories
PMoH: Palestinian Ministry of Health
SE: Scale Efficiency
UNRWA: United Nations Relief and Works Agency for Palestine Refugees in the Near East
VRS: Variable Returns to Scale
WHO: World Health Organization
